# *In Vitro* and *In Situ* Activity-Based Labeling of Fibroblast Activation Protein with UAMC1110-Derived Probes

**DOI:** 10.3389/fchem.2021.640566

**Published:** 2021-04-14

**Authors:** Yentl Van Rymenant, Muhammet Tanc, Roos Van Elzen, An Bracke, Olivier De Wever, Koen Augustyns, Anne-Marie Lambeir, Mark Kockx, Ingrid De Meester, Pieter Van Der Veken

**Affiliations:** ^1^ Laboratory of Medical Biochemistry, Department of Pharmaceutical Sciences, University of Antwerp, Antwerp, Belgium; ^2^ Laboratory of Medicinal Chemistry, Department of Pharmaceutical Sciences, University of Antwerp, Antwerp, Belgium; ^3^ HistoGeneX, Antwerp, Belgium; ^4^ Laboratory of Experimental Cancer Research, Faculty of Medicine and Health Sciences, University of Ghent, Ghent, Belgium

**Keywords:** fibroblast activation protein, activity-based probe, fluorophore, biomarker, histochemistry

## Abstract

Fibroblast activation protein (FAP) is a proline-selective protease that belongs to the S9 family of serine proteases. It is typically highly expressed in the tumor microenvironment (TME) and especially in cancer-associated fibroblasts, the main cell components of the tumor stroma. The exact role of its enzymatic activity in the TME remains largely unknown. Hence, tools that enable selective, activity-based visualization of FAP within the TME can help to unravel FAP’s function. We describe the synthesis, biochemical characterization, and application of three different activity-based probes (biotin-, Cy3-, and Cy5-labeled) based on the FAP-inhibitor UAMC1110, an in-house developed molecule considered to be the most potent and selective FAP inhibitor available. We demonstrate that the three probes have subnanomolar FAP affinity and pronounced selectivity with respect to the related S9 family members. Furthermore, we report that the fluorescent Cy3- and Cy5-labeled probes are capable of selectively detecting FAP in a cellular context, making these chemical probes highly suitable for further biological studies. Moreover, proof of concept is provided for *in situ* FAP activity staining in patient-derived cryosections of urothelial tumors.

## Introduction

Fibroblast activation protein (FAP; EC3.4.21. B28), also called seprase, belongs to the S9 family of serine proteases. Other members of this family include dipeptidyl peptidases 4, 8, and 9 (DPP4, DPP8, and DPP9) and prolyl oligopeptidase (PREP) (Rawlings et al., 2018). FAP is a post-prolyl proteolytic enzyme with endopeptidase as well as dipeptidyl peptidase activity, both exerted by the same active site composed of Ser^624^-Asp^702^-His^734^ (O’Brien and O’Connor, 2008). FAP is expressed as a homodimeric transmembrane protein or as a soluble plasma protease (antiplasmin cleaving enzyme, APCE) (Goldstein et al., 1997; Piñeiro-Sánchez et al., 1997; Lee et al., 2006).

Typically, FAP is highly expressed in cancer-associated fibroblasts (CAFs) in 90% of all epithelial tumors, whereas its expression is low to undetectable in most healthy adult tissues (Garin-Chesa et al., 1990). Besides its expression in the tumor stroma, FAP expression is also upregulated in certain malignant tumor cells, such as glioblastoma (Busek et al., 2016), breast (Kelly et al., 1998), cervical (Jin et al., 2003), pancreatic (Shi et al., 2012), and colorectal (Iwasa et al., 2003) cancer cells. Taken together, it has been proven that FAP is a potential target for cancer diagnostics and therapeutics. Moreover, FAP expression is also increased in several nonmalignant diseases where active tissue remodeling is involved, including hepatic fibrosis (Levy et al., 1999), idiopathic pulmonary fibrosis (Acharya et al., 2006), atherosclerosis (Brokopp et al., 2011), rheumatoid arthritis (Bauer et al., 2006), and myocardial infarction (Tillmanns et al., 2015).

Nevertheless, the main focus of FAP research lies within the field of oncology. Elevated FAP expression and/or activity has been detected in multiple human cancers and serves as a negative prognostic marker for the overall survival time and progression of the disease (Henry et al., 2007; Zhang et al., 2007; Cohen et al., 2008; Ju et al., 2009). Furthermore, it has been proven that FAP^+^CAFs contribute to tumor progression, resistance to immunotherapy, and metastasis (Zi et al., 2015). FAP’s endopeptidase activity (gelatinase activity) plays a major role in remodeling of the extracellular matrix, which is an important mechanism in the invasion and metastasis of the cancer cells (Monsky et al., 1994; Lee et al., 2011). Additionally, its enzymatic activity is also reported to have an angiogenesis promoting effect (Huang et al., 2004). Lately, it was suggested that FAP also contributes to immunosuppression in the tumor microenvironment (TME). Firstly, Chen et al. found that CAFs with high FAP expression are responsible for the induction of immune checkpoint blockade resistance in a colorectal cancer mouse model (Chen et al., 2017). Secondly, Feig et al. demonstrated that the depletion of FAP^+^CAFs resulted in immune control of pancreatic ductal adenocarcinoma tumors (Feig et al., 2013). More recently, it was reported that FAP promotes immunosuppression through upregulation of CCL2, resulting in increased tumor growth by enhancing recruitment of myeloid-derived suppressor cells and tumor-associated macrophages (Yang et al., 2016; Lin et al., 2019).

In contrast to the above findings, other studies demonstrate that elevated FAP expression is correlated with tumor suppression instead of tumor promotion (Ariga et al., 2001; Ramirez-Montagut et al., 2004). Hence, it is suggested that the role of FAP in cancer is likely to depend on cell context and tumor microenvironment.

Despite progress in understanding FAP biology, the enzyme’s exact role in the TME remains largely unknown, in particular, whether and how FAP’s proteolytic activity might be involved in this context. Interestingly, most of the previous studies investigating FAP’s function in cancer are relying on FAP mRNA and/or protein expression rather than on the investigation of FAP’s enzymatic activity. Quantification of the latter could therefore offer alternative and/or complementary information, especially because several studies have reported a statistically relevant link between the enzymatic activity of FAP and disease severity and progression (Cheng et al., 2005; Puré and Blomberg, 2018). Therefore, activity-based probes (ABPs) for FAP have the potential to unveil several functions of FAP in the complex tumor environment. Moreover, these tools could also offer value in FAP biomarker studies. Noteworthy, high FAP-potency and selectivity towards the related enzymes are crucial for these ABPs because the other S9 proteases are ubiquitously expressed in human cells and tissues.

**FIGURE 1:**
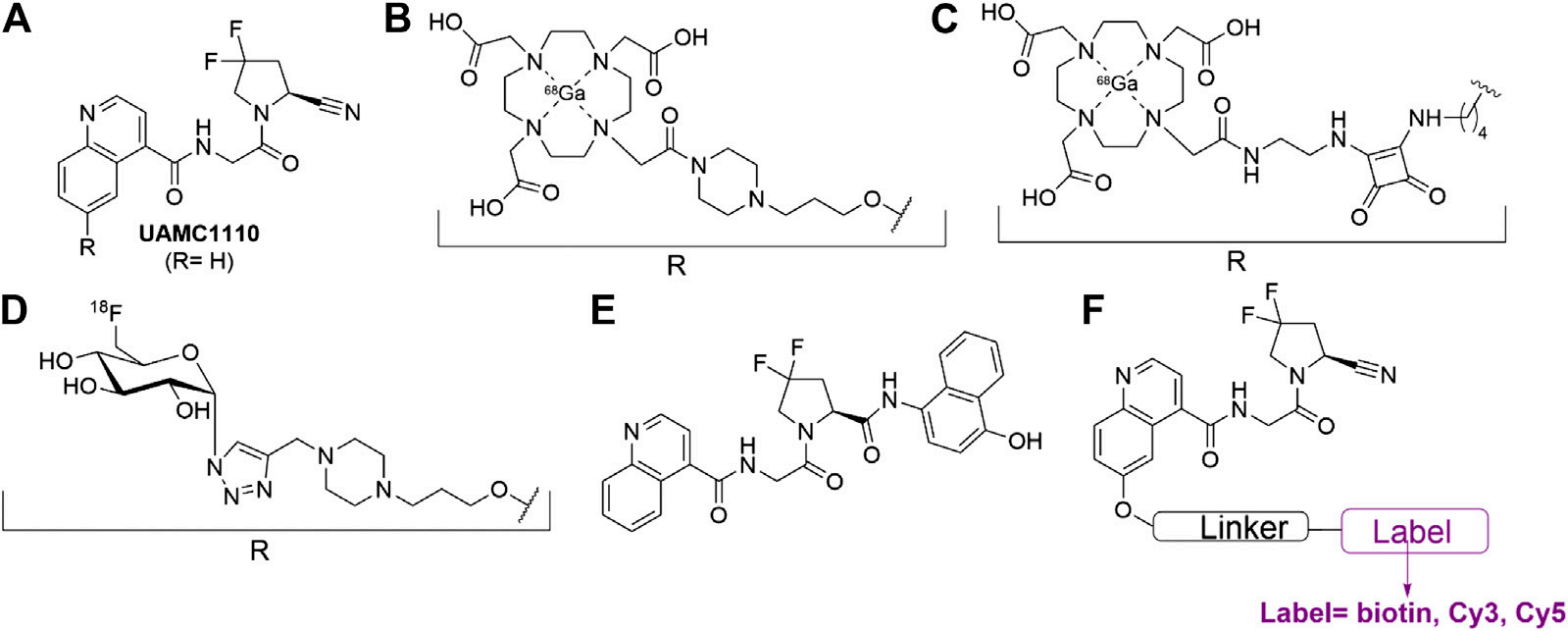
FAP-inhibitor UAMC1110 and examples of UAMC1110 derivatives that have been reported as activity-based probes (ABPs) (Jansen et al., 2014; Lindner et al., 2018; De Decker et al., 2019; Loktev et al., 2019; Moon et al., 2020; Toms et al., 2020). **(A)** Parent compound UAMC1110 (Jansen et al., 2014). **(B)** Gallium-68 labeled radiotracer reported in 2018 (Lindner et al., 2018). **(C)** Gallium-68 labeled radiotracer reported in 2020 (Moon et al., 2020). **(D)** Fluorine-18 labeled radiotracer reported in 2019 (Toms et al., 2020). **(E)** Designed, selective FAP substrate reported in 2019 (De Decker et al., 2019). **(F)** UAMC1110-derived probes, synthesized for this study.

Earlier efforts from our groups delivered UAMC1110 (Figure 1A), the most potent and selective, orally bioavailable FAP inhibitor reported to date (Ryabtsova et al., 2012; Jansen et al., 2013; Jansen et al., 2014). Both we and others have used this molecule as a main structural element of FAP-targeted ABPs (Dvorakova et al., 2017; Simon et al., 2018; Bracke et al., 2019; Zhang et al., 2020). Important examples include FAP probes for PET/CT imaging and even radiotherapeutic applications (Figure 1B–D) (Lindner et al., 2018; Loktev et al., 2019; Toms et al., 2020; Kratochwil et al., 2019; Luo et al., 2020; Moon et al., 2020). While these molecules might have unprecedented potential for *in vivo* imaging of FAP-positive tumors, their radioactivity impedes implementation for biomarker applications outside the field of molecular imaging. In this respect, ABPs with fluorometric or colorimetric read-out can be expected to be highly relevant for these other domains of biomarker research. Two types of compounds can be pursued: 1) fluorogenic or colorigenic substrates of FAP and 2) FAP-inhibitor-derived molecules equipped with a fluorophore, a chromophore, or another label type that can be quantified via standard molecular biology technology. Recently, our research groups reported several UAMC1110-derived FAP substrates with excellent cleavage efficiencies and selectivities (De Decker et al., 2019) compared to other reported FAP-processed probes (Figure 1E) (Bainbridge et al., 2017; Poplawski et al., 2013; Li et al., 2012; Keane et al., 2013). Here, we present FAP-inhibiting ABPs that contain a UAMC1110 moiety, equipped with either a biotin, a Cy3, or a Cy5 moiety (Figure 1F). The choice to introduce the label at the 6-position of the quinoline ring was based on structure-activity relationship research that we published earlier (Jansen et al., 2014). Likewise, several other published UAMC1110-derived ABPs (shown, e.g., in Figure 1) also indicated that large groups are accepted at this position without taking significant affinity penalties (Figure 1).

Noteworthy, other fluorescent ABPs targeting FAP have been reported very recently by Roy et al. (2020). It concerns conjugates of a small molecule FAP inhibitor with either FITC or a near-infrared fluorescent cyanine dye. The small molecule inhibitor used as the basis for these molecules was claimed by the authors to be FAP-selective, however, without providing experimental details. The authors verified the selectivity of the derived probes by using an *in vitro* fluorescence binding assay with FAP-transfected HEK293T cells. This method, nonetheless, does not constitute a reliable validation of selectivity with respect to the other members of the S9 enzyme family. Furthermore, Konvalinka and coworkers published two types of UAMC1110-derived ABPs both equipped with biotin or the fluorophore ATTO488 (Dvorakova et al., 2017; Simon et al., 2018). The first type consisted of polymer-bound, multivalent probes called “iBodies”. These molecules showed high selectivity towards recombinant DPP4, DPP9, and PREP based on IC_50_ experiments. Separately, structurally distinct compounds obtained via a stochastic photomodification approach were obtained. For the latter, only FAP-Ki values were reported, but there were no selectivity data towards the other related family members. Nonetheless, these compounds did not show aspecific staining in FAP-negative cells, indicating *in situ* selectivity at least under the experimental conditions used (Simon et al., 2018).

To support further investigation of FAP’s enzymatic function in several pathological conditions, we decided to prepare three different ABPs (biotin-, Cy3-, and Cy5-labeled) based on the FAP-inhibitor UAMC1110. In order to provide a benchmark status to these molecules for biomarker applications, unequivocal characterization data are included with respect to target affinity, selectivity, binding characteristics, and applications *in cellulo* and *in situ*.

## Materials and Methods

### Chemistry

**SCHEME 1:**
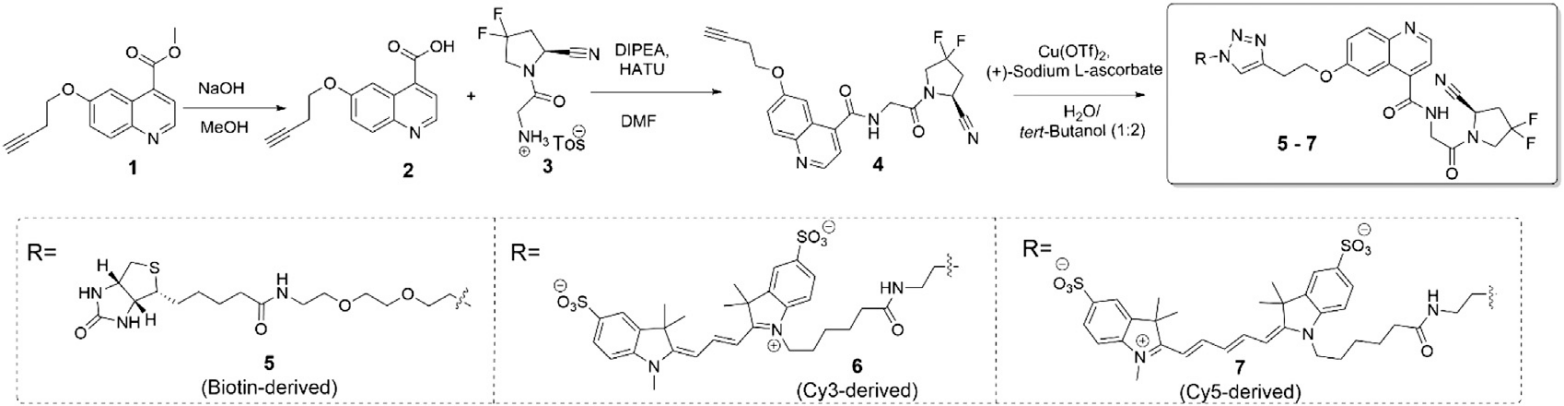
Overview of the synthetic preparation of target compounds **5**–**7**.

The synthesis of the target compounds is summarized in Scheme 1 **.** The alkyne-derived quinoline-4-carboxylate **1**, obtained relying on a procedure by Toms et al., was used as the starting material (Poplawski et al., 2013). It was coupled with *N*-glycyl-(4,4-difluoropyrrolidine-2-carbonitrile), delivering intermediate **4**, the common precursor to the desired probe molecules. The latter were obtained via copper-assisted azide-alkyne click ligation of **4** and the corresponding, azide-derived biotin-, Cy3-, or Cy5-derived labels. The latter were acquired from commercial sources. The biotin-, Cy3-, and Cy5-labeled probes are referred to as compounds **5**, **6**, and **7**, respectively. Detailed procedures, yields, and compound characterization data are provided in the Supporting Information file.

### Enzymes

Recombinant human FAP (rhFAP, extracellular domain, amino acid 27-760) with a C-terminal His-tag was expressed and purified in Sf9 insect cells as described by Moon et al. (Moon et al., 2020). Human dipeptidyl peptidase 4 (DPP4) was purified from human seminal plasma as described before (De Meester et al., 1996). Human recombinant dipeptidyl peptidases 8 and 9 (DPP8 and DPP9) were expressed in Sf9 insect cells using the N-terminal BaculoDirect insect cell expression system (Invitrogen) and were purified as published before (De Decker et al., 2019). Human recombinant prolyl oligopeptidase (PREP) was expressed in BL21 (DE3) cells and purified as described by De Decker et al. (2019).

### Cell Culture, Transfection, and Stimulation

Human embryonic kidney cells (HEK293T) were purchased from ATCC and were cultured in Dulbecco’s modified Eagle’s medium (DMEM) supplemented with 10% FBS, 100 U/ml penicillin, and 100 μg/ml streptomycin. Telomerase immortalized colon cancer-associated fibroblasts (hTERT CT5.3 CAFs) were cultured in DMEM supplemented with 10% FCS, 2 mM l-glutamine, 100 U/ml penicillin, and 100 μg/ml streptomycin (De Vlieghere et al., 2015). Both cell lines were maintained in a humidified incubator at 37°C with 5% CO_2_.

Transfection of HEK293T cells with FAP or an empty vector (mock-transfection) was performed as described earlier (De Decker et al., 2019). Briefly, HEK293T cells were transiently transfected at 70–80% confluency with the pDEST40-hFAP vector (encoding the full ORF of FAP) or pDEST40-empty vector (Thermo Fisher) using the Lipofectamine 2000 transfection reagent (Thermo Fisher) in a 1:3 ratio of DNA/Lipofectamine 2000. The HEK293T cells transfected with the pDEST40-empty vector (mock-transfection) were used as a negative control (De Decker et al., 2019). Cells were used 48 h after transfection for the western blotting and/or (immuno)fluorescence experiments.

Recombinant human TGF-β1 (PeproTech; 100–21-10UG) was used for stimulation of CAFs. Briefly, 0.2 × 10^6^ CAFs were seeded in a T25 flask and incubated for 24 h. Subsequently, the cells were stimulated with 10 ng/ml TGF-β1 for 4 days at 37°C in a 5% CO_2_ environment. Stimulated CAFs were used for activity-based probe experiments.

### Determination of IC_50_ for Fibroblast Activation Protein, Dipeptidyl Peptidases 4, 8, and 9, and Prolyl Oligopeptidase

IC_50_ values of **5**–**7** (biotin-, Cy3-, and Cy5-labeled ABPs) for FAP and PREP were determined as described by Moon et al*.* (Moon et al., 2020). IC_50_ measurements for DPP4, DPP8, and DPP9 were performed analogously using H-Gly-Pro-AMC as the substrate at the respective final concentrations of 65 µM (with DPP4) and 100 µM (with DPP8/DPP9) at pH 7.6 (0.1 M Tris-HCl buffer with 0.1 M NaCl and 0.1 mg/ml BSA). For all enzymes, the methods and data fitting were performed as published by Moon et al*.* (Moon et al., 2020). Experiments were repeated at least in triplicate, and the results are represented as an average ± SD.

### Reactivity of the Probes with Fibroblast Activation Protein and Related Peptidases Based on SDS-PAGE Analysis

To confirm the selectivity of Cy3-labeled **6** and Cy5-labeled **7**, 100 nM enzyme was preincubated for 15 min at 37°C. Subsequently, the enzymes were incubated for 20 min at 37°C with various concentrations of either Cy3 or Cy5 ABP (2.5, 0.5, 0.1, 0.02, and 0 µM) in, respectively, FAP assay buffer (50 mM Tris-HCl pH 8.0, 140 mM NaCl), DPP assay buffer (0.1 M Tris-HCl buffer with 0.1 M NaCl pH 7.6), or PREP assay buffer (0.1 MK-phosphate, 1 mM EDTA, 1 mM DTT pH 7.4). After 20 min incubation, the samples were boiled in 4x reducing SDS-PAGE loading buffer for 5 min and loaded on a 7.5% separation gel. The gels were run for 1 h at 140 V with the PageRuler™ Plus Prestained Protein marker (Life Technologies). Afterward, the Cy5-labeled gels were directly scanned at the red 700 nm (red channel for Cy5) channel of the Odyssey Sa fluorescence imaging system (Li-COR), and the images were analyzed using the Image Studio software (version 5.2). For the Cy3 experiments, the gels were scanned at the green channel (520 nm, Cy3 probe) and red channel (630 nm, molecular weight marker) using the Amersham 600 RGB imager, and image acquisition was performed with the ImageJ software.

### Reversibility of the Probes Based on a Dialysis Experiment

Recombinant human FAP (rhFAP) was incubated for 15 min at 37°C with a concentration of probe that was predicted to inhibit around 90% of FAP’s activity (**5**: 1.08 nM; **6**: 2.50 nM; **7**: 1.35 nM; UAMC1110: 0.77 nM diluted in FAP assay buffer: 50 mM Tris-HCl pH 8.0, 140 mM NaCl, and 1 mg/ml BSA). As a solvent control, rhFAP was incubated with 0.0002% DMSO. After 15 min of incubation, FAP activity was determined as published by Bracke et al. (2019). Subsequently, the samples were dialyzed at 4°C against FAP assay buffer (using a 10 kDa cut-off Slide-A-Lyzer MINI dialysis device (Thermo Fisher). Buffer (14 ml) was exchanged after 3 h, 6 h, 24 h, 3 days, and 7 days, and after each of these time points, a FAP activity measurement was performed. For the UAMC1110 parent compound, FAP activity was only measured on days 3 and 7.

### Fibroblast Activation Protein Labeling in Cell Lysates Using the Cy5 Probe by SDS-PAGE and Western Blotting

HEK293T cells were transfected with pDEST40-hFAP or pDEST40-empty vector in T25 flasks following the protocol described in *Cell Culture, Transfection, and Stimulation*. After 48 h, cells were washed 3 times with DPBS followed by harvesting using a cell scraper. Cells were centrifuged at 250 x g for 5 min at 4°C. TGF-β1-stimulated CAFs were harvested using a nonenzymatic dissociation solution (Sigma-Aldrich) and centrifuged at 125 x g for 5 min at 4°C.

The harvested cells were lysed in western blot lysis buffer (1% Triton X-100, 150 mM NaCl, 5 mM EDTA, and a complete protease inhibitor cocktail tablet [Roche Diagnostics] in a 50 mM Tris buffer, pH 7.5) for 1 h with frequent agitation and centrifuged at 12,000 × *g* for 10 min at 4°C. The supernatant was used for western blot analysis. Protein concentration was determined using the Bradford protein quantification assay. 20 µg of HEK293T lysates and 50 µg of CAF lysate were incubated with 200 nM of Cy5-labeled **7** for 20 min at 37°C. Subsequently, the samples were diluted in 4x SDS-PAGE sample buffer, boiled, and subjected to SDS-PAGE (7.5% acrylamide gels, 140 V, 1 h) followed by protein transfer onto a low fluorescence PVDF membrane (Bio-Rad, 250 mA, 1 h). Membranes were cut into two above 50 kDa. Subsequently, the membranes were blocked with 2.5% BSA in TBS-T for 1 h at room temperature followed by overnight incubation at 4°C with a primary antibody against FAP (Rabbit anti-FAP, Abcam, Ab207178, 1:1,000 diluted in 1% BSA in TBS-T). *β*-Actin was used as a loading control (mouse anti-β-actin, Sigma, A1978, 1:10,000 diluted in 1% BSA in TBS-T). Afterward, the membranes were incubated with fluorescent secondary antibodies (IRDye® 800CW, goat anti-rabbit, and IRDye® 800 CW donkey anti-mouse, LI-COR, 1:10,000 in 1% BSA in TBS-T) for 1 h at room temperature. Between the different incubations, the membranes were washed 5 × 5 min with TBS‐T. Membranes were afterward simultaneously scanned at an excitation of 685 nm (Cy5-labeled probe 7; 700 nm channel) and 785 nm (secondary antibody; 800 nm channel) using the odyssey Sa fluorescence imaging system (LI-COR). Images were analyzed using the Image Studio software (version 5.2).

### *In Situ* Detection of Fibroblast Activation Protein Expression and/or Activity in Fibroblast Activation Protein-Transfected HEK293T by (Immuno)fluorescence

The day before transfection, 80,000 cells HEK293T were seeded into 8-well Nunc Lab-Tek II CC2 Chamber Slides (Life Technologies). After 24 h, the cells were transfected with pDEST40-hFAP or pDESR40-empty vector using the protocol described in *Cell Culture, Transfection, and Stimulation*. After transfection, the HEK293T cells were washed twice with DPBS followed by incubation with 500 nM of either Cy3-labeled **6** or Cy5-labeled **7** (diluted in Opti-MEM) for 2 h at 37°C in the dark. Next, cells were washed twice with DPBS followed by fixation with 4% paraformaldehyde (PFA) for 30 min at room temperature. The PFA was gently removed, and the fixed cells were then washed twice with DPBS followed by a blocking step in 5% BSA for 1 h at room temperature. Subsequently, the cells were incubated with the anti-FAP F19 monoclonal antibody (purified in-house; 1.43 mg/ml; 1:500 in 3% BSA in DPBS) overnight at 4°C in the absence of light. The antibody was removed, and the cells were washed twice with DPBS followed by incubation with a secondary antibody (Alexa Fluor 488 Goat anti-Mouse, A11001, Life Technologies) for 1 h at room temperature in the absence of light and a similar washing procedure. The slides were mounted using Vectashield antifade mounting medium with DAPI (Vector lab, H-1200).

For the confocal microscopy analysis, an inverted Leica TCS SP8 confocal laser scanning microscope was used. DAPI was detected by the DAPI channel (405 nm), and the Cy3 probe and Cy5 probe were detected with a white light laser (WLL) at the respective wavelengths of 554 and 645 nm. The secondary antibody (Alexa Fluor 488) was visualized with a WLL at 495 nm. Images were made with 63x objective and 1.5x zoom and analyzed using the ImageJ software. All images were taken under the same settings.

### Proof of Concept for Detection of *In Situ* Fibroblast Activation Protein Activity in Fibroblast Activation Protein-Positive Urothelial Cancer Cryosections

Fresh frozen urothelial cancer tissue was purchased from BioIVT (patient ID: ILS51040FT2). The tissue was optimal cutting temperature (OCT) compound embedded, and cryosections of 5 µm were made using a Leica CM1950 cryostat. Samples were stored at –80°C until use.

The urothelial cryosections were thawed at room temperature, and thereafter the tissue was lined with an IHC PAP pen (Enzo Life Sciences). The lined tissue sections were then washed 2 times for 5 min with DPBS, followed by two washes (5 min) with 50 mM Tris-HCl pH 7.4, 140 mM NaCl. Subsequently, the sections were incubated with either probe **6** or **7** (1 µM in FAP assay buffer, 200 µL) for 2 h at 37°C in a humidified chamber in the dark. After washing (3 × 5 min with DPBS), the sections were fixed with 4% paraformaldehyde (PFA) for 10 min at room temperature. Next, sections were washed three times and the slides were mounted as described above.

Images were obtained using a Zeiss Axio imager. DAPI was detected using the DAPI channel, and the Cy3 and Cy5 probe were detected with a Colibri 7 LED light source at the respective excitation wavelengths of 548 and 650 nm. Images were made using a 20× objective, and image analysis was performed using the ImageJ software.

## Results and Discussion

### Fibroblast Activation Protein Affinity and Selectivity of Probes 5–7

**FIGURE 2:**
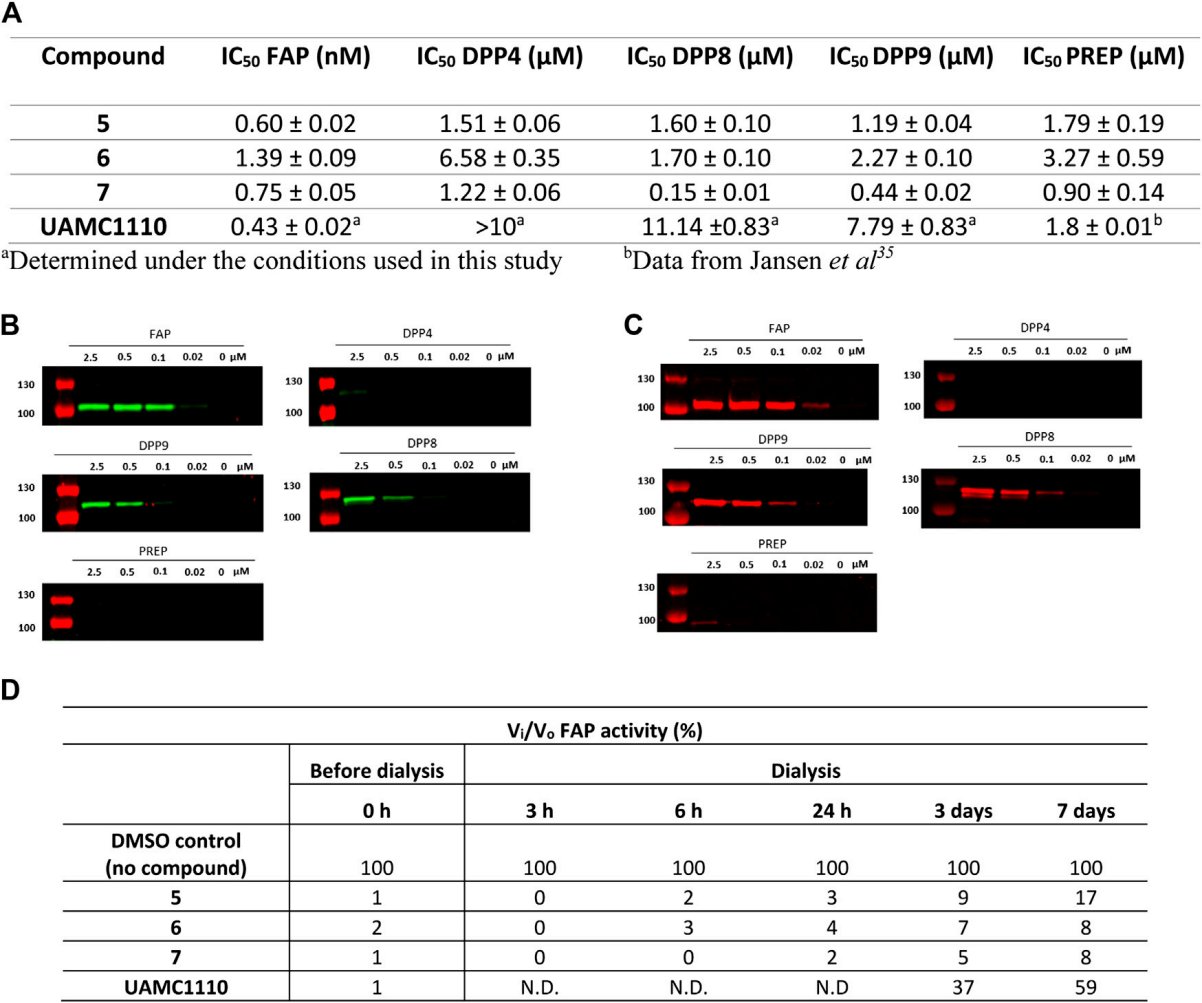
Potency, selectivity, and irreversibility of the ABPs (**5**, **6**, and **7**) for FAP. **(A)** IC_50_ values of **5–7** (biotin-, Cy3-, and Cy5-activity-based probe) toward FAP and related peptidases (DPP4, DPP8, DPP9, and PREP). Experiments were carried out in triplicate (*n* = 3), and results are displayed as mean ± SD. Labeling of recombinant FAP, DPP4, DPP8, DPP9, and PREP using **6** (Cy3-labeled) **(B)** and **7** (Cy5-labeled) **(C)** probes. Enzymes (100 nM final concentration) were incubated separately with various probe concentrations for 20 min and then subjected to SDS-PAGE analysis. **(D)** Dialysis experiment to investigate reversibility of the developed probes (*n* = 2). N.D.: not determined.

All three ABPs were characterized for their potency toward FAP and selectivity toward the related peptidases (DPP4, DPP8, DPP9, and PREP). The results are displayed in Figure 2A. Parent compound UAMC1110 was used as a reference compound. Compared to Jansen et al., a slightly higher potency for UAMC1110 toward FAP is reported here (0.43 vs. 3.2 nM). The latter is due to the introduction of a new measurement method using a lower concentration of human recombinant FAP (Jansen et al., 2014).

All three ABPs exhibited subnanomolar affinity toward human recombinant FAP, within the same order of magnitude as the parent compound (Figure 2A). These results provide additional evidence that derivatization of UAMC1110 at the 6-position of the quinoline ring tolerates the introduction of diverse and sterically large groups.

IC_50_ values for DPP4, DPP8, DPP9, and PREP are in the low micromolar range, corresponding to ∼1000-fold selectivity for **5** and **6**. Nevertheless, under the experimental conditions used here, compound **7** (the Cy5 probe) exhibits some cross-reactivity with DPP8 and DPP9 (Figure 2A).

To confirm the selectivity of probes **5**–**7**, an *in vitro* labeling experiment using recombinant FAP, DPP4, DPP8, DPP9, and PREP was performed. The latter consisted of preincubating different probe concentrations with 100 nM of the respective enzymes, after which the mixtures were submitted to SDS-PAGE electrophoresis and, ultimately, detection of labeled enzymes. For biotin-labeled **5**, a streptavidin-HRP-based method was used for visualization of labeled enzymes (Supplementary Method 2.1). This however did not result in significant staining (data not shown). Although we do not have a clear explanation for this, we assume that steric hindrance could be involved here. More specifically, it is possible that when **5** is bound to FAP, the biotin label is buried in FAP’s structure, resulting in steric hindrance that prevents binding to streptavidin. Conversely, proteins labeled with fluorescent **6** and **7** were easily detected at the fluorophores’ respective wavelengths of maximum fluorescence (*λ*_max_) (Figures 2B,C). Nonetheless, it should be mentioned that, due to the differences in *λ*_max_ values for both fluorophores, it was required to use two different instruments for detecting **6** and **7** during the SDS-PAGE experiment. Therefore, a direct comparison of sensitivity under experimental conditions is difficult. In general, the observed fluorescent labeling experiments reflect the selectivity of the probes determined in the IC_50_ experiments. Fluorescent FAP labeling with **7** (Cy5-labeled probe) is visible down to 20 nM probe concentration, whereas with **6** (Cy3-labeled probe), FAP can be visualized down to 100 nM probe concentration. Since FAP’s concentration in the experiment is 100 nM, this corresponds, respectively, to a 1:1 (Cy3) and 0.2:1 (Cy5) probe/enzyme concentration ratio. Furthermore, almost no cross-reactivity is observed with DPP4 and PREP, while some cross-reactivity with DPP8 and DPP9 can be observed for both the Cy3- and Cy5-based molecule, again approximately proportional to the IC_50_ values of these compounds (Figures 2B,C). Overall, it is surprising that cross-reactivity toward DPP8/9 is observed in SDS-PAGE. This finding suggests a “tight binding” profile, despite the relatively lower inhibitory potencies of 6 and 7 toward DPP8/9. Nonetheless, it is also worth mentioning that an overall lower potency does not per se preclude the formation of a covalent bond between the active site serine residue and the carbonitrile warhead of **6** and **7**. Structural studies (e.g., X-ray diffraction of [inhibitor-protease] complexes) could shed more light on this issue. Full gel scans of **6** (Cy3) and **7** (Cy5) in gel scanning experiments are displayed in Supplementary Figure S1, S2.

That we were able to detect the complex of FAP with the fluorescent Cy3 and Cy5 probes after performing SDS-PAGE shows that the compounds bind very tightly to FAP’s active site. To rationalize this observation, a covalent interaction of the carbonitrile warhead in these compounds and FAP’s catalytic serine residue can reasonably be assumed. This leads to the formation of an imidate adduct, as suggested by earlier Structure-Activity-Relationship (SAR) studies that we have performed on the class of inhibitors to which parent compound UAMC1110 belongs (Jansen et al., 2014). Comparably, crystallographic evidence for imidate formation has been delivered for structurally related inhibitors of DPP4 (Berger et al., 2018). Nonetheless, this covalent interaction is known to be transient and reversible, and we have earlier also established the reversible, covalent binding profile of the probes’ parent compound UAMC1110 (Jansen et al., 2014). To further investigate the probes’ binding profile, a dialysis experiment was performed (Figure 2D). The results of the latter indeed confirm that when FAP was incubated with the probes followed by extensive dialysis, the recovery of FAP activity is more limited compared to the parent compound UAMC1110. We conclude therefore that **6** and **7** practically behave as irreversible inhibitors of FAP within the conditions and the timeframes of our experiments. These observations were in line with the previously published FAP-targeting iBody report. Here, no k_off_-rates using surface plasmon resonance (SPR) could be measured (Dvorakova et al., 2017).

Furthermore, using classical dilution-based enzyme kinetics experiments, no activity recovery could be detected, also indicating very low k_off_-rates. In summary, these probes possess a remarkable “tight binding” profile, in which the [ligand-target] complex is significantly more stable than any other carbonitrile-based inhibitor that we have investigated to date. It cannot be excluded that other moieties of the probes than the carbonitrile warhead alone are involved in this process. Although cyanine-based dyes have not been reported to covalently label proteins, it cannot be excluded that in these compounds they contribute to binding via other physicochemical interactions with FAP (e.g., ionic interactions with the sulfonate groups).

### Selective Labeling of Fibroblast Activation Protein with Probe 7 (Cy5-Labeled) in Cell Lysates

**FIGURE 3:**
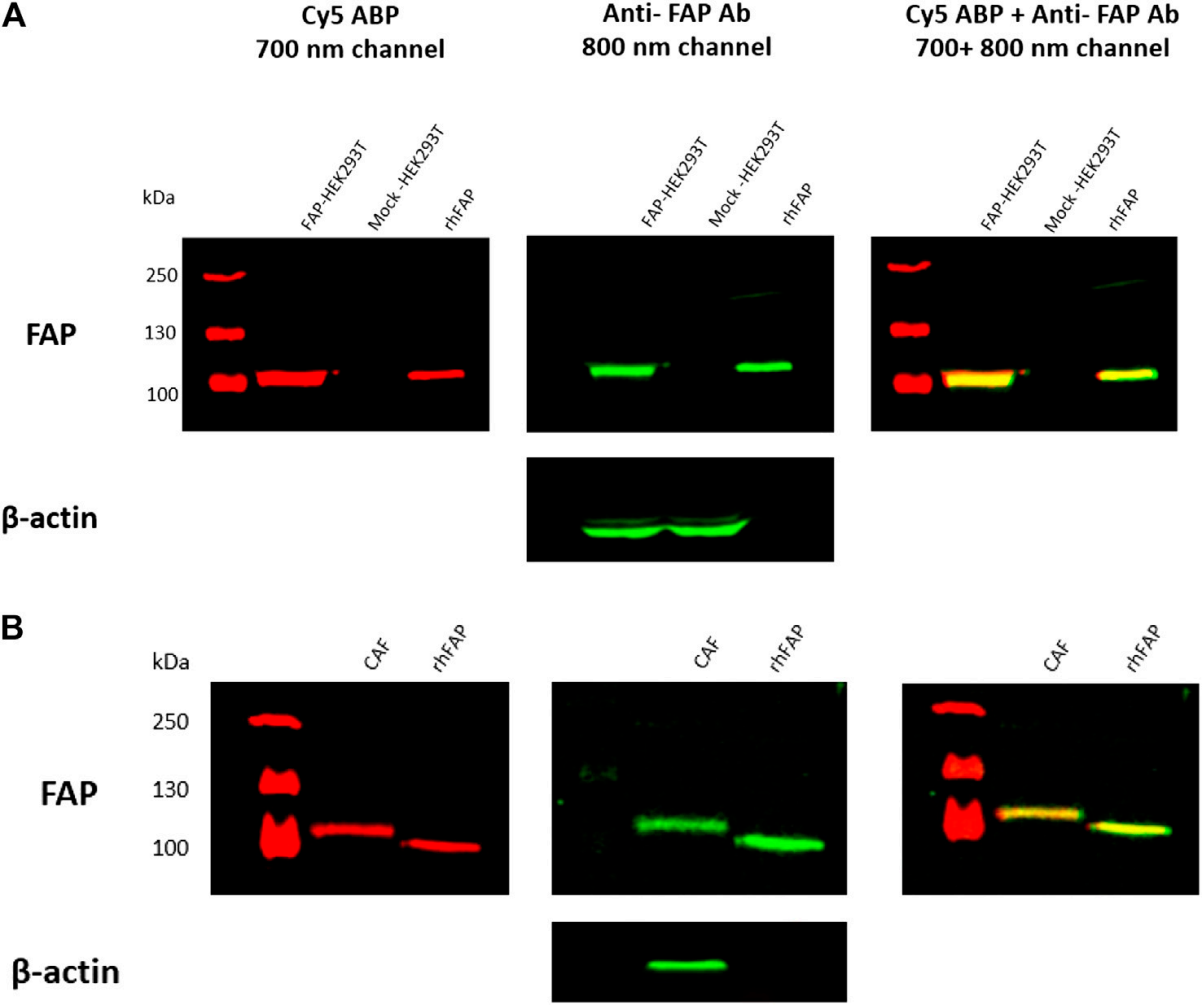
FAP labeling in FAP-transfected HEK293T **(A)** and TGF-β1-stimulated CAF cells **(B)** using compound 7 (Cy5-labeled probe). Cell lysates were incubated with 200 nM of **7** (Cy5 probe) for 30 min at 37°C, followed by SDS-PAGE and western blot analysis. The red bands (700 nm channel) correspond to FAP labeling with the Cy5 probe, whereas the green bands (800 nm channel) correspond to FAP labeling with a selective anti-FAP antibody. *β*-Actin was used as loading control for FAP- and mock-transfected HEK293T cells. In‐house purified rhFAP was loaded as positive control. The Cy5 probe is selective towards FAP in both the HEK293T **(A)** and CAF cell lines **(B)**, as confirmed by co‐labeling of the Cy5 probe (red) with the anti‐FAP antibody (green).

Based on the IC_50_ and the in-gel fluorescence SDS-PAGE experiments, we concluded that Cy5-labeled **7** was the most potent probe toward FAP. Because **7** showed most cross-reactivity with other S9 enzyme family members, we investigated whether this probe can be used to selectively label active FAP in cell lysates. To this end, we prepared lysates from FAP- and mock-transfected HEK293T cells and TGF-β1-stimulated CAFs. Cell lysates were incubated with 200 nM of the Cy5 probe, and the mixtures were subjected to SDS-PAGE, followed by western blot analysis. The results demonstrated that **7** (red) can selectively label overexpressing and endogenous FAP in the cell lysates, as confirmed by colabeling with the anti-FAP antibody (green) (Figures 3A,B). No labeling of FAP in the mock-transfected cells is observed, again confirming selective labeling. To demonstrate the endogenous DPP8/9 expression/activity pattern in FAP- and mock-transfected HEK293T cells and CAFs, a western blotting experiment and DPP8/9 activity measurement were performed (Supplementary Figure S3). This experiment indicated that **7** displayed a high selectivity toward FAP, under the reaction conditions used in this experiment. Remarkably, in both the FAP-transfected HEK293T lysates and the CAF lysates, the Cy5 probe seems to label FAP at equal sensitivity compared to the anti-FAP antibody, implying that this probe can be used as an alternative for antibodies in the detection of active FAP in a biological context.

Given that Cy5-labeled **7** could selectively label FAP in these lysates, we assume that also the Cy3 probe is capable of doing so, especially because of its comparable FAP affinity and higher selectivity toward DPP8 and DPP9. Nevertheless, performing similar experiments in the future may be necessary to prove this hypothesis.

### *In Situ* Detection of Active Fibroblast Activation Protein by Fluorescence Microscopy in Fibroblast Activation Protein-Transfected HEK293T Cells

Next, we used both **6** and **7** to detect FAP activity in HEK293T-transfected cells by fluorescence microscopy.

**FIGURE 4:**
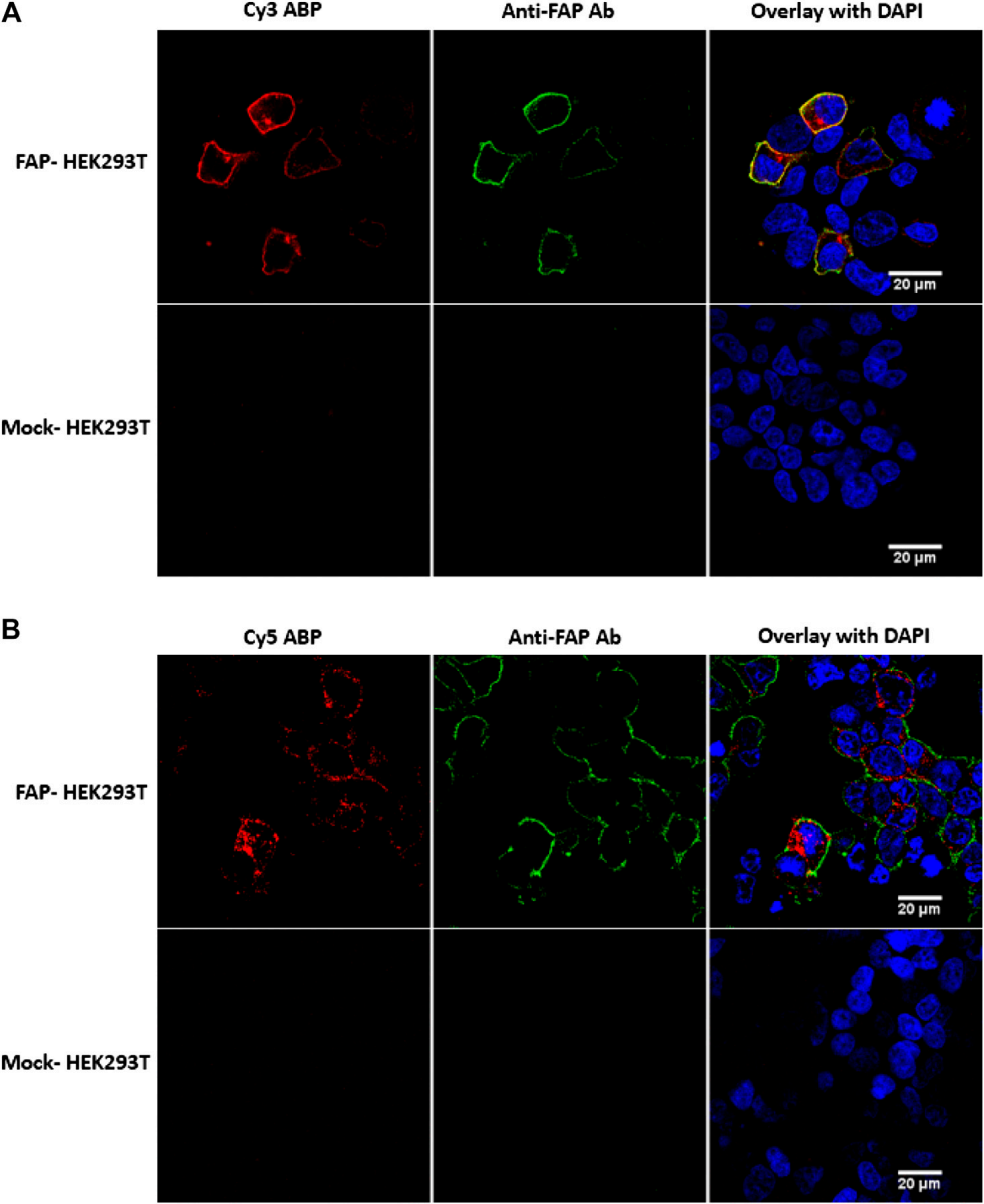
FAP activity labeling with probes **6** (Cy3-labeled) and **7** (Cy5-labeled) in FAP-transfected HEK293T cells. **(A)** Clear membrane colocalization of the Cy3 probe (red) and the F19 anti-FAP antibody (green), indicating selectivity of the fluorescent Cy3 probe. **(B)** The Cy5 probe caused cytoplasmatic staining, whereas the antibody is mainly visible at the membrane, suggesting an internalization of the FAP-Cy5 complex. No aspecific stainings were visible in the mock-transfected HEK293T cells. Images are representative of at least two independent experiments (*n* = 2).

With the Cy3-labeled **6**, a clear membrane-bound FAP staining was visible on the FAP-transfected HEK cells (Figure 4A). The selectivity of **6** was confirmed by colabeling of FAP with this ABP (red) and the monoclonal F19 anti-FAP antibody (green). Mock-transfected HEK293T cells were used as a negative control and again proved that no aspecific staining was present for both the Cy3 probe and the antibody.

In contrast to **6**, the Cy5-labeled **7** caused a cytoplasmatic staining rather than a membrane staining (Figure 4B). However, there is only Cy5 cytoplasmatic staining visible in the HEK293T cells that were successfully transfected with pDEST40-hFAP. In addition, no Cy5 staining was visible at the mock-transfected HEK293T cells. Given that HEK293T cells endogenously express DPP8 and DPP9 and no staining is visible in the mock-transfected cells, we conclude that the Cy5 probe specifically binds to FAP. To rationalize this observation, an internalization of FAP upon binding with the Cy5 probe can be assumed. This is in line with a previously published study that demonstrated FAP internalization upon binding with an ATTO488 conjugated FAP-iBody, also based on the parent UAMC1110 compound (Dvorakova et al., 2017). Furthermore, it is also known that the F19 monoclonal anti-FAP antibody does not initiate FAP internalization, not even at 37°C (Fischer et al., 2012). Nonetheless, for a more precise explanation for Cy5-FAP complex internalization in contrast to Cy3-FAP membrane staining, more mechanistic studies are needed.

With biotin-labeled **5**, a similar *in situ* staining experiment in FAP-transfected HEK293T cells was performed following the method described in the Supplementary Material. Unfortunately, we were not able to visualize FAP on the cells using the biotin-labeled probe (data not shown). This is in line with the observations during the western blotting experiments and again could indicate possible steric hindrance for binding with streptavidin. Hence, no further experiments with biotin-labeled 5 were performed.

### Proof of Concept for Detection of Active Fibroblast Activation Protein in Human Urothelial Bladder Cancer Using Probes 6 and 7

**FIGURE 5:**
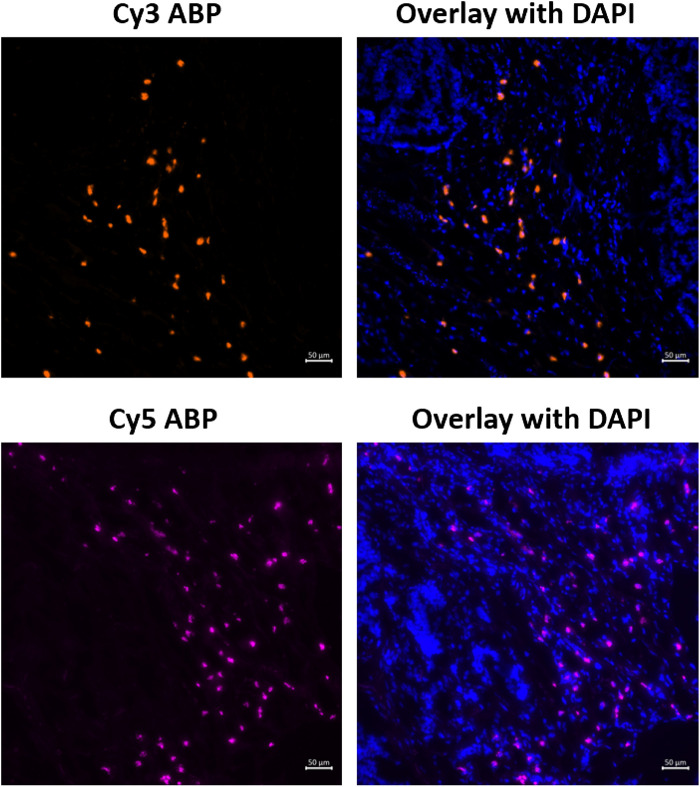
Staining of active FAP in the tumor microenvironment of urothelial cancer sections using 6 (Cy3-probe) and 7 (Cy5-probe). Images are representative of at least three different tissue sections for each activity-based probe (*n* = 3).

To investigate **6** and **7**’s ability to label active FAP in the tumor stroma, both were used to stain human urothelial cancer cryosections. A clear staining of FAP activity is visible for both **6** and **7** under the conditions used in this experiment (Figure 5). Based on morphology, the FAP staining is clearly restricted to the tumor stroma, whereas almost no staining was visible in the tumor cells themselves, indicating selective labeling since FAP is only known to be expressed in the tumor stroma of urothelial cancer (whole slide image, Supplementary Figure S4) (Mezheyeuski et al., 2020).

## Conclusion

In this work, we developed three FAP-specific activity-based probes (biotin-, Cy3-, and Cy5-labeled) with a subnanomolar affinity toward FAP and pronounced selectivity toward the related S9 family members. Moreover, it was concluded that the ABPs bind very tightly to the active site of FAP, resulting in the practically irreversible character of the compounds. We further demonstrated that Cy5-labeled **7** could selectively label not only overexpressed but also endogenous FAP in cell lysates. Finally, we showed the applicability of both the Cy3-labeled **6** and Cy5-labeled **7** for *in situ* FAP labeling in cells and cancer cryosections.

Unfortunately, we were not able to visualize FAP labeled with biotin-based probe **5**, in neither the Western blotting experiments nor the *in situ* staining of FAP-transfected HEK293T cells. In the future, investigating FAP-specific biotin probes with a longer linker may lead to more promising results.

Consequently, the newly developed ABPs can be considered as useful chemical tools that have several advantages compared to the classical immunochemical techniques (e.g., immunofluorescence). First of all, staining of FAP in cells and/or tissues is much faster using fluorescent ABPs compared to general antibody-relying techniques, resulting in significant time savings. Secondly, ABPs are much smaller than antibodies, making it possible to use ABPs for *in situ* protein interaction and colocalization studies. Where larger antibodies can cause steric hindrance in such studies, small-molecule ABPs could be very suitable for accurate protein-interaction experiments.

In summary, we have developed FAP-selective fluorescent ABPs, which allow accurate detection of active FAP in cells and tissue cryosections. The developed FAP-selective ABPs can be used to facilitate the understanding of FAP’s enzymatic activity in the tumor microenvironment. Finally, given the importance of FAP not only in the pathophysiology of cancer but also in several other diseases (idiopathic pulmonary fibrosis, rheumatoid arthritis, atherosclerosis, hepatic fibrosis, etc.), the applicability of these newly developed probes could be extended to unravel FAP’s role in these diseases as well.

## Data Availability

The original contributions presented in the study are included in the article/Supplementary Material; further inquiries can be directed to the corresponding authors.

## Abbreviations

ABP, activity-based probe; DPP, dipeptidyl peptidase; FAP, fibroblast activation protein; rhFAP, recombinant human FAP; PREP, prolyl oligopeptidase; Cy3, cyanine 3; Cy5; cyanine 5; CAF, cancer-associated fibroblast; TME, tumor microenvironment; PFA, paraformaldehyde; WLL, white light laser.
