# Supplementary material for: In Vitro and In Situ Activity-Based Labeling of Fibroblast Activation Protein with UAMC1110-Derived Probes
Source: Front Chem. 2021 Apr 14;9:640566. doi: 10.3389/fchem.2021.640566 (PMC8114891; doi:10.3389/fchem.2021.640566)
Supplement: Supplementary file 1 [file datasheet1.docx]

Supplementary Material and Results

**1. Material and methods**

All reagents and Anhydrous solvents and were purchased from Acros Chemicals, Sigma–Aldrich, Fluorochem and TCI chemicals. All reactions involving air- or moisture- sensitive compounds were performed under a nitrogen atmosphere using dried glassware and syringes. Synthesized compounds were characterized with 1H-NMR, 13C-NMR and mass spectrometry. NMR spectra (1H NMR, 13C NMR, DEPT-135, DEPT-90, HSQC, HMBC) were recorded on a 400 MHz Bruker Avance DRX-400 spectrometer. Chemical shifts are expressed in parts per million (ppm) and coupling constants (J) are in Hertz (Hz). Splitting patterns are mentioned as follows: s, singlet; d, doublet; sept, septet; t, triplet; q, quadruplet; m, multiplet; br s, broad singlet; dd, double of doublet, appt, apparent triplet, appq, apparent quartet, br t broad triplet. ES mass spectra were obtained from an Esquire 3000plus ion trap mass spectrometer from Bruker Daltonics. Purity was determined using two diverse HPLC systems using, respectively, a mass and UV detector. Water (A) and CH3CN (B) were used as eluents. LC-MS spectra were recorded on an Agilent 1100 Series HPLC system using an Alltech Prevail C18 column (2.1 × 50 mm, 3 µm) coupled with an Esquire 3000plus as MS detector and a 5-100% B, 20 min gradient was used with a flow rate from 0.2 mL/min. Formic acid 0.1% was added to solvents A and B. Waters acquity UPLC system coupled to a waters TQD ESI mass spectrometer and waters TUV detector was used. A waters acquity UPLC BEH C18 1.7 μm 2.1 x 50 mm column was used. Solvent A: water with 0.1% formic acid, solvent B: acetonitrile with 0.1% formic acid. Method I: 0.15 min 95% A, 5% B then in 1.85 min from 95% A, 5% B to 95% B, 5% A, then 0.25 min (0.350 mL/min), 95% B, 5% A. The wavelength for UV detection was 254 nm. Method II: flow 0.4 mL/min, 0.25 min 95% A, 5% B, then in 4.75 min to 95% B, 5% A, then 0.25 min 95% B, 5% A, followed by 0.75 min 95% A, 5% B. The wavelength for UV detection was 214 nm. Where necessary flash purification was performed on a Biotage® ISOLERA One flash system equipped with internal variable dual-wavelength diode array detector (200-400 nm). SNAP cartridges (4-50 g) were used. Gradients used varied by purification.

**
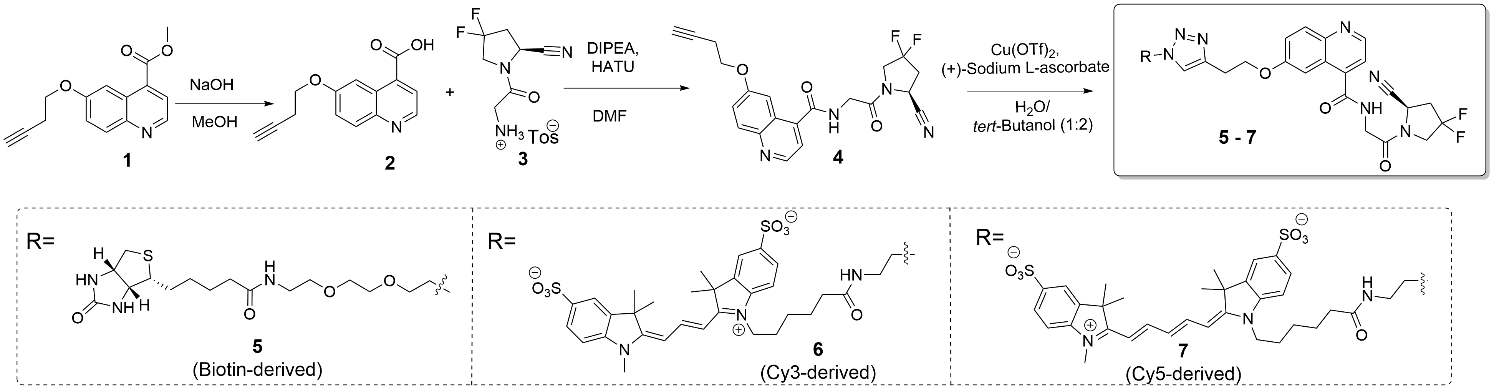
1.1 General procedure for the synthesis of compounds**

**Scheme 1: Synthesis of target compounds.**

**6-(but-3-yn-1-yloxy)quinoline-4-carboxylic acid (2)**

Methyl 6-(but-3-yn-1-yloxy) quinoline-4-carboxylate, **1**, was prepared according to the literature as alkylation of quinoline with corresponding alkyne (48 % yield). *^1^*^,2^

**1H NMR** (400 MHz, DMSO-*d6*, δ): 2.74 (td, *J*= 2.75, 6.47,6.47 Hz, 2H), 2.95 (t, *J* = 2.54, 1H), 3.97 (s, 3H), 4.20 (t, *J*= 6.86 Hz, 2H), 7.52 (dd, *J* = 2.84, 9.35 Hz, 1H), 7.93 (d, *J*= 4.45 Hz, 1H), 8.03 (d, *J* = 9.25 Hz, 1H), 8.06 (d, *J* = 4.45 Hz, 1H), 8.89 (d, *J*= 4.45 Hz, 1H); **13C NMR** (100 MHz, DMSO-*d6*, δ): 18.78, 52.81, 66.15, 72.66, 81.26, 104.34, 122.30, 122.69, 125.57, 131.40, 132.60, 144.84, 147.67, 157.46, 166.27. **MS** t_R_ 1.67 min, MS (ESI) m/z 256.2 [M+H] (89 %)

For 6-(but-3-yn-1-yloxy)quinoline-4-carboxylic acid, **2**, a 10 mL round bottom flask was charged with methyl 6-(but-3-yn-1-yloxy)quinoline-4-carboxylate (0,12 g, 0.470 mmol) NaOH (0.038 g, 2eq) and MeOH (4 mL). Afterwards 0.5 mL water was added and reaction mixture stirred for 1 hour at at 40 ℃. After reaction completed (UPLC monitoring), it was diluted with 5 mL of water at room temperature. Organic impurities extracted with 1 mL of EtOAc and aqueous phase acidify until pH 5 with 1 M HCl. Formed precipitate was collected by filtration through a glass filter and dried under *vacuo*. The titled compound was obtained as white solid (49 % yield).

**1H NMR** (400 MHz, DMSO-*d6*, δ): 2.74 (td, *J*= 2.63, 6.45,6.56 Hz, 2H), 2.95 (t, *J* = 2.59, 1H), 4.20 (t, *J*= 6.46 Hz, 2H), 7.52 (dd, *J* = 2.83, 9.28 Hz, 1H), 7.94 (d, *J*= 4.42 Hz, 1H), 8.04 (d, *J* = 9.20 Hz, 1H), 8.19 (d, *J* = 2.78 Hz, 1H), 8.88 (d, *J*= 8.89 Hz, 1H), 13.81 (s, 1H); **13C NMR** (100 MHz, DMSO-*d6*, δ): 18.82, 66.09, 72.64, 85.52, 104.95, 121.92, 122.46, 126.00, 131.20, 144.45, 147.77, 157.04, 166.74. **MS** t_R_ 0.23 min, MS (ESI) m/z 240.1 [M-H] (100 %)

**(S)-6-(but-3-yn-1-yloxy)-N-(2-(2-cyano-4,4-difluoropyrrolidin-1-yl)-2-oxoethyl)quinoline-4-carboxamide (4)**

(S)-2-(2-cyano-4,4-difluoropyrrolidin-1-yl)-2-oxoethan-1-aminium 4-methylbenzenesulfonate ,**3**, was prepared according to the literature, and used as it is for the next step *^2^*.

A 10 mL round bottom flask was charged with 6-(but-3-yn-1-yloxy)quinoline-4-carboxylic acid (0,052 g, 0.216 mmol), **2**, and 5 mL dry DMF then O-(7-Azabenzotriazol-1-yl)-N,N,N',N'-tetramethyluroniumPF6 (0.090 g, 1.1 eq) and *N,N*-Di-isopropylethylamine (0.188 ml, 5eq) were added and the reaction stirred for 10 min. Following (S)-2-(2-cyano-4,4-difluoropyrrolidin-1-yl)-2-oxoethan-1-aminium 4-methylbenzenesulfonate (0.109 g, 1.4eq ) added. After reaction completed (UPLC monitoring) all volatiles was removed *via* rotavap. Following the crude diluted with 5 mL DCM and washed with 1 mL 1 M aqueous HCl (x3) and 1 mL water (x3). Combined organic layers was dried over sodium sulfate and eliminated under reduced pressure *via* rotavap. The crude purified with flash chromatography (0-6 % MeOH/DCM). The titled compound was lyophilized and obtained as white powder (90 % yield).

**1H NMR** (400 MHz, DMSO-*d6*, δ): 2.73 (td, *J*= 2.57, 6.33, 6.51 Hz, 2H), 2.90 (m, 3H), 4.24 (m, 6H), 5.15 (dd, *J* = 2.67, 4.93 Hz, 1H), 7.48 (dd, *J* = 2.74, 4.51 Hz, 1H), 7.52 (d, *J*= 4.22 Hz, 1H), 7.90 (d, *J*= 2.82 Hz, 1H), 7.99 (d, *J* = 9.15 Hz, 1H), 8.81 (d, *J* = 4.37 Hz, 1H), 9.11 (t, *J*= 5.95 Hz, 1H); **13C NMR** (100 MHz, DMSO-*d6*, δ): 19.15, 31.77, 36.78, 41.82, 44.69, 44.75, 66.69, 72.90, 81.91 105.10, 118.25, 119.70, 122.93, 125.79, 131.30, 141.32, 144.62, 148.10, 157.03, 167.97, 168.49. **MS** t_R_ 0.23 min, UPLC MS (ESI) m/z 413.2 [M+H]^+^ (94 %)

***N*-(2-((*R*)-2-cyano-4,4-difluoropyrrolidin-1-yl)-2-oxoethyl)-6-(2-(1-(2-(2-(2-(5-((3a*R*,4*R*,6a*S*)-2-oxohexahydro-1*H*-thieno[3,4-*d*]imidazol-4-yl)pentanamido)ethoxy)ethoxy)ethyl)-1*H*-1,2,3-triazol-4-yl)ethoxy)quinoline-4-carboxamide (5)**

A 5 mL glass vial charged with *N*-(2-(2-(2-azidoethoxy) ethoxy) ethyl)-5-((3a*S*,4*S*,6a*R*)-2-oxohexahydro-1*H*-thieno[3,4-d] imidazol-4-yl) pentanamide (0.017 g, 0.042 mmol), (*S*)-6-(but-3-yn-1-yloxy)-*N*-(2-(2-cyano-4,4-difluoropyrrolidin-1-yl)-2-oxoethyl) quinoline-4-carboxamide (0,021g, 1.2 eq), and trifluoromethanesulfonic acid copper salt (0.015 g, 1 eq) and 1 mL of t-BuOH. Afterwards, sodium *L*-ascorbate (0.034 g, 5 eq) in 0.05 mL water was added to mixture. After consumption of the starting compounds (UPLC monitoring) reaction diluted with 1 mL of water and 0.2 mL of EtOAc and stirred for a minute. Yellow precipitate was collected by filtration and purified by flash column chromatography on reversed phase C-18 silica gel using 5-50 % H_2_O/ACN. The titled compound was obtained as white solid (30 % yield).

**1H NMR** (400 MHz, DMSO-*d6*, δ): 1.30 (m, 2H), 1.39-1.63 (m, 4H), 2.04 (t, *J* = 7.54 Hz, 2H), 2.56 (d, *J* = 11.96 Hz, 1H), 2.73-2.96 (m, 3H), 3.06 (m, 1H), 3.15 (m, 3H), 3.33 (t, *J* = 5.67 Hz, 2H), 3.45 (m, 1H), 3.50 (m, 1H), 3.79 (t, *J* = 5.04 Hz, 2H), 4.06-4.33 (m, 8H), 4.40 (t, *J* = 6.93 Hz, 2H), 4.49 (t, *J* = 5.09 Hz, 2H), 5.13 (d, *J* = 9.44 Hz, 2H), 6.37 (brs, 1H), 6.43 (brs, 1H), 7.46 (d, *J* = 9.44 Hz, 1H), 7.53 (d, *J* = 4.41 Hz, 1H), 7.82 (t, *J* = 5.67 Hz, 1H), 7.90 (s, 1H), 7.95 (s, 1H), 7.98 (d, *J* = 9.38 Hz, 1H), 8.82 (brs, 1H), 9.12 (t, *J* = 6.11 Hz, 1H); **13C NMR** (100 MHz, DMSO-*d6*, δ): 19.14, 25.7, 25.8, 28.4, 28.7, 29.5, 35.5, 38.0, 38.8, 41.8, 49.7, 55.9, 59.6, 61.5, 66.7, 67.4, 67.5, 69.2, 69.6, 69.8, 69.9, 72.9, 76.4, 105.0, 105.1, 118.2, 119.7, 123.1, 123.6, 131.0, 141.4, 144.4, 147.8, 157.32, 163.2, 167.9, 168.4, **MS** t_R_ 0.29 min, UPLC MS (ESI) 0.5 m/z 407.5 [M+2H]^+^ , m/z 813.5 [M+H]^+^, m/z 835.5 [M+Na]^+^, m/z 857.5 [M+HCOO]^-^ (85%)

**1-(6-((3-(4-(2-((4-((2-((*R*)-2-cyano-4,4-difluoropyrrolidin-1-yl)-2-oxoethyl)carbamoyl)quinolin-6-yl)oxy)ethyl)-1*H*-1,2,3-triazol-1-yl)propyl)amino)-6-oxohexyl)-3,3-dimethyl-2-((*E*)-3-((*E*)-1,3,3-trimethyl-5-sulfonatoindolin-2-ylidene)prop-1-en-1-yl)-3*H*-indol-1-ium-5-sulfonate (6)**

A 5 mL glass vial charged with potassium 1-(6-((3-azidopropyl) amino)-6-oxohexyl)-3,3-dimethyl-2-((*E*)-3-((*E*)-1,3,3-trimethyl-5-sulfonatoindolin-2-ylidene) prop-1-en-1-yl)-3*H*-indol-1-ium-5-sulfonate (0.022 g, 0.030 mmol), (*S*)-6-(but-3-yn-1-yloxy)-*N*-(2-(2-cyano-4,4-difluoropyrrolidin-1-yl)-2-oxoethyl) quinoline-4-carboxamide (0,015g, 1.2 eq), and trifluoromethanesulfonic acid copper salt (0.011 g, 1 eq) and 1 mL of t-BuOH. Afterwards sodium *L*-ascorbate (0.024 g, 5 eq) in 0.05 mL water was added to mixture. After consumption of the starting compounds (UPLC monitoring). All volatiles were eliminated under *vacuo.* Residue purified by flash column chromatography on reversed phase C-18 silica gel using 5-50 % H_2_O/ACN. The titled compound was obtained as red solid (35 % yield).

**1H NMR** (400 MHz, DMSO-*d6*, δ): 1.21-1.32 (m, 5H), 1.52-1.59 (m, 3H), 1.68 (s, 12H), 1.86 (t, *J* = 6.89 Hz, 1H), 2.06 (t, *J* = 7.35 Hz, 1H), 2.78-2.93 (m, 2H), 2.99 (q, *J* = 5.89, 7.78 Hz, 2H), 3.17 (m, 2H), 3.63 (s, 3H), 4.08-4.39 (m, 10H), 5.13 (d, *J* = 6.50 Hz, 1H), 6.45 (d, *J* = 7.94 Hz, 1H), 6.49 (d, *J* = 7.70 Hz, 1H), 6.63 (m, 0.5H), 6.94 (m, 0.5H), 7.38 (d, *J* = 8.04 Hz, 1H), 7.48-7.53 (m, 2H), 7.65-7.68 (m, 2H), 7.80 (s, 2H), 7.86-7.91 (m, 2H), 7.80 (s, 1H), 8.33 (t, *J* = 13.64 Hz, 1H), 9.10 (t, *J* = 6.03 Hz, 1H); **13C NMR** (100 MHz, DMSO-*d6*, δ): 25.4, 25.8, 26.2, 27.2, 27.7, 27.9, 29.6, 30.4, 30.8, 31.8, 31.9, 35.6, 36.2, 41.8, 44.7, 47.6, 49.4, 61.2, 67.5, 103.2, 103.8, 105.1, 111.1, 115.4, 115.5, 118.2, 120.2, 120.3, 123.1, 123.3, 125.8, 127.4, 129.9, 130.0, 130.5, 132.6, 140.5, 141.1, 142.4, 143.0, 143.9, 144.6, 146.1, 146.3, 147.9, 150.2, 155.3, 155.8 157.3, 167.9, 168.5, 172.5, 174.5, 175.34; **MS** t_R_ 2.39 min, UPLC MS (ESI) 0.5 m/z 556.5 [M+2H]^+^ , m/z 1111.6 [M+H]^+^, m/z 1155.8 [M+2Na-H]^+^, m/z 857.5 [M+HCOO]^-^ , 0.5 m/z 556.5 [M-2H]^-^, m/z 1109.7 [M-H]^-^ (100 %)

**1-(6-((3-(4-(2-((4-((2-((S)-2-cyano-4,4-difluoropyrrolidin-1-yl)-2-oxoethyl)carbamoyl)quinolin-6-yl)oxy)ethyl)-1H-1,2,3-triazol-1-yl)propyl)amino)-6-oxohexyl)-3,3-dimethyl-2-((1E,3E)-5-((E)-1,3,3-trimethyl-5-sulfonatoindolin-2-ylidene)penta-1,3-dien-1-yl)-3H-indol-1-ium-5-sulfonate (7)**

A 5 mL glass vial charged with potassium 1-(6-((3-azidopropyl)amino)-6-oxohexyl)-3,3-dimethyl-2-((1*E*,3*E*)-5-((*E*)-1,3,3-trimethyl-5-sulfonatoindolin-2-ylidene)penta-1,3-dien-1-yl)-3H-indol-1-ium-5-sulfonate (0,015 g, 0.020 mmol), (*S*)-6-(but-3-yn-1-yloxy)-*N*-(2-(2-cyano-4,4-difluoropyrrolidin-1-yl)-2-oxoethyl)quinoline-4-carboxamide (0,012g, 1.5 eq), and trifluoromethanesulfonic acid copper salt (0.007 g, 1 eq) and 1 mL of t-BuOH. Afterwards sodium *L*-ascorbate (0.016 g, 5 eq) in 0.05 mL water was added to mixture. After consumption of the starting compounds (UPLC monitoring). All volatiles were eliminated under *vacuo.* Residue purified by flash column chromatography on reversed phase C-18 silica gel using 5-50 % H_2_O/MeOH. The titled compound was obtained as blue solid (22 % yield).

**1H NMR** (400 MHz, DMSO-*d6*, δ): 1.21-1.32 (m, 7H), 1.51-1.57 (m, 3H), 1.68 (s, 12H), 1.86 (t, *J* = 7.05 Hz, 1H), 1.97-2.09 (m, 2H), 2.81-2.87 (m, 1H), 2.99 (q, *J* = 7.02, 7.76 Hz, 2H), 3.17 (t, *J* = 4.80 Hz, 2H), 3.58 (s, 3H), 4.08-4.25 (m, 6H), 4.30 (t, *J* = 7.32 Hz, 2H), 4.39 (t, *J* = 5.75 Hz, 2H), 5.13 (d, *J* = 6.49 Hz, 1H), 6.25 (d, *J* = 13.56 Hz, 1H), 6.30 (d, *J* = 13.56 Hz, 1H), 6.55 (t, *J* = 12.49 Hz, 1H), 6.30 (d, *J* = 13.56 Hz, 1H), 7.30 (d, *J* = 8.57 Hz, 1H), 7.46 (dd, *J* = 2.86, 5.35 Hz, 1H), 7.50 (d, *J* = 4.28 Hz, 1H), 7.61-7.64 (m, 2H), 7.81 (s, 1H), 7.86 (t, *J* = 5.75 Hz, 1H), 7.89 (d, *J* = 2.87 Hz, 1H), 7.97 (d, *J* = 9.77 Hz, 1H), 8.00 (s, 1H), 8.36 (t, *J* = 13.22 Hz, 1H), 8.81 (brs, 1H), 9.10 (t, *J* = 5.42 Hz, 1H); **13C NMR** (100 MHz, DMSO-*d6*, δ): 22.8, 23.8, 25.3, 25.8, 26.1, 27.3, 27.6, 29.5, 30.4, 31.7, 34.1, 34.7, 35.5, 36.2, 38.6, 41.8, 44.6, 47.6, 48.9, 49.3, 66.1, 67.5, 105.1, 110.6, 115.3, 115.4, 115.5, 116.1, 116.3, 118.2, 119.3, 123.0, 123.3, 125.9, 127.3, 128.1, 128.5, 129.9, 130.0, 132.6, 140.8, 140.9, 141.15, 142.5, 143.2, 144.6, 145.6, 145.8, 154.6, 155.8, 157.3, 167.9, 168.5, 172.5; **MS** t_R_ 2.53 min, UPLC MS (ESI) 0.5 m/z 569.5 [M+2H]^+^ , m/z 1137.6 [M+H]^+^, m/z 857.5 [M+HCOO]^-^ , 0.5 m/z 567.5 [M-2H]^-^, m/z 1136.4 [M-H]^-^ (100%)

**1.2 Detection of FAP labeled with 5 (biotin-labeled ABP) using streptavidin-HRP and Western blotting**

Recombinant human FAP (100 nM) was pre-incubated for 15 min at 37 °C. Subsequently, FAP was incubated for 20 minutes at 37 °C with various concentrations of either the Biotin-labeled ABP (2.5 µM, 0.5 µM, 0.1 µM, 0.02 µM and 0 µM). After 20 min incubation, the samples were boiled in 4 X reducing SDS-PAGE loading buffer for 5 minutes followed by loading on a 7.5% separation gel. After electrophoresis, biotin-labeled FAP was transferred onto a nitrocellulose membrane (250 mA, 1 hour). Subsequently, the membranes were blocked with 2.5% BSA in TBS-T for 1 hour at room temperature, followed by 1 hour incubation with streptavidin-HRP (1 : 200 in TBST, R&D) at room temperature. Between different incubation periods, the membrane was washed 5 x 5 minutes with TBS-T. The membrane was developed using the West Femto Maximum Sensitivity Substrate (Life Technologies) and visualized using the OptiGo imager with Proxima AQ-4 software (Isogen Life Sciences).

**1.3 Detection of *in situ* FAP in FAP transfected HEK293T cells using biotin-based probe 5**

HEK293T cells were seeded and transfected as described in the main material and methods section 2.8. After transfection, HEK293T cells were incubated with 500 nM of biotin-labeled **5** (diluted in Opti-MEM) for 2 hours at 37 °C. Next, cells were washed twice with DPBS followed by fixation with 4% paraformaldehyde (PFA) for 30 minutes at room temperature. The PFA was gently removed and the fixed cells were then washed twice with DPBS followed by an incubation step with Streptavidin-Alexa Fluor 488 (S32354, Life Technologies, 1:200 in DPBS) for 1 hour at room temperature in the absence of light. Slides were mounted as described in the main material and methods section 2.8.

**1.4 DPP8/9 expression pattern in FAP- and Mock-transfected HEK293T cells and western blotting**

DPP8/9 Expression: Twenty µg of HEK293T lysates, 50 µg of CAF lysate and rhDPP8/9 (20 nM) were diluted in 4 x SDS-PAGE sample buffer, boiled and were subjected to SDS-PAGE (7.5% acrylamide gels, 140 V, 1 hour) followed by protein transfer onto a nitrocellulose membrane (BioRad, 250 mA, 1 hour). Membranes were cut into two above 50 kDa. Subsequently, the membranes were blocked with 5% milk powder in TBS-T for 1 hour at room temperature followed by overnight incubation at 4 °C with a primary antibody against DPP8 (Rabbit anti-DPP8, Abcam, Ab42075, 1 : 1000 diluted in blocking buffer) or DPP9 (Mouse anti-DPP, OriGene, Ta504307, 1/1000 in blocking buffer). β-actin was used as loading control (Mouse anti-β-actin, Sigma, A1978, 1 : 10 000 diluted in blocking buffer). Afterwards, the membranes were incubated with secondary antibodies (Goat-anti-Rabbit IgG- HRP (1/5000, 65-6120, Invitrogen) and Goat anti-mouse- IgG-HRP (1/4000, A4416, Sigma) for 2 hours at room temperature. Between the different incubations, the membranes were washed 5 x 5 minutes with TBST. Detection was performed using the Supersignal West Femto Maximum Sensitivity Substrate (ThermoFisher) and images were analyzed using the Proxima AS-4 software (IsoGen LifeSciences).

**DPP8/9 activity:**  DPP8/9 activity was measured in HEK293T and CAF lysates using our previously published in-house developed method. *^3, 4^* Gly-Pro-4-Methoxy-β-naphtylamide was used as the substrate (500 µM final concentration) and 1G244 as DPP8/9 inhibitor. DPP8/9 activity was normalized to the protein content in the lysates by Bradford quantification.

**2. Results**


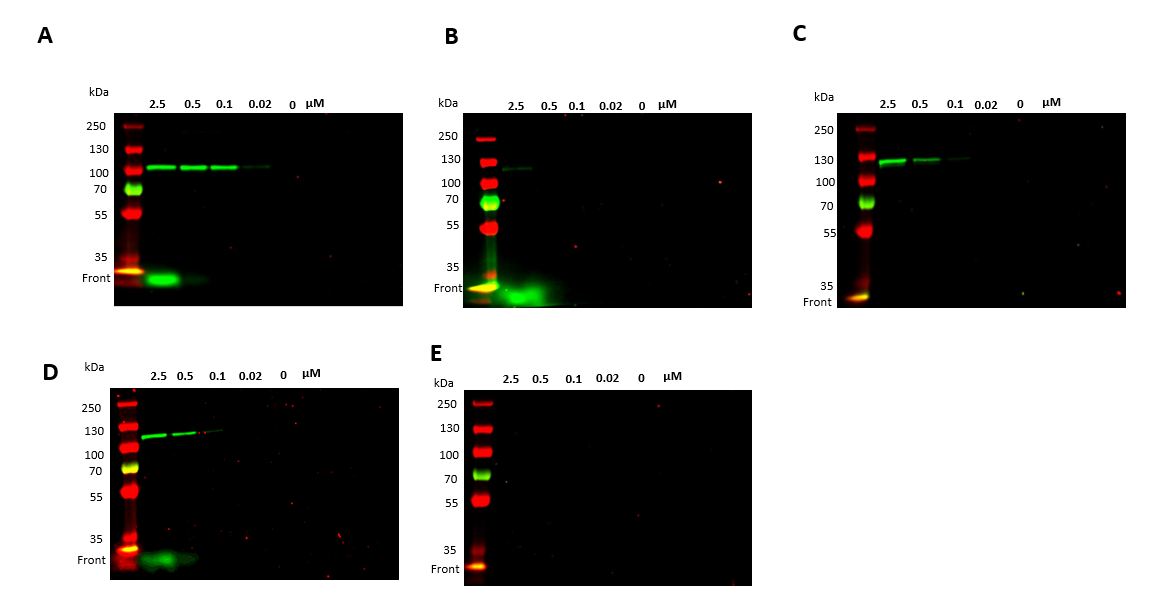
**2.1 Selectivity of the Cy3- and Cy5- labeled probes based on SDS-PAGE analysis**


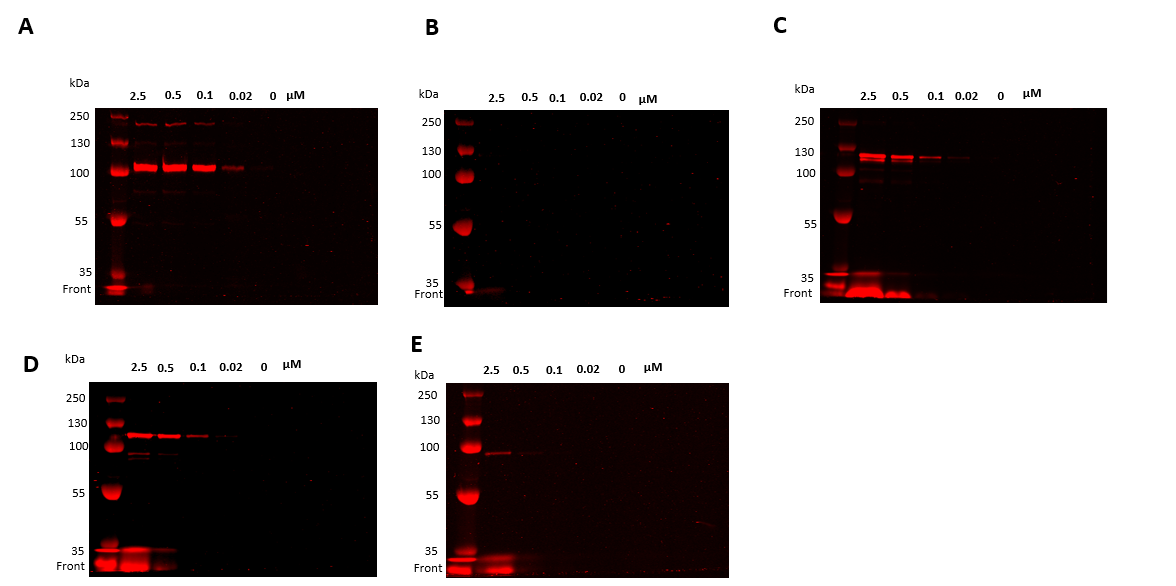
**Supplementary figure 1S**: Full gel scans of SDS-PAGE analysis with the Cy3 probe **(A)** FAP, **(B)** DPP4, **(C)** DPP8, **(D)** DPP9, **(E)** PREP

**Supplementary figure 2S**: Full gel scans of SDS-PAGE analysis with the Cy5 probe **(A)** FAP, **(B)** DPP4, **(C)** DPP8, **(D)** DPP9, **(E)** PREP


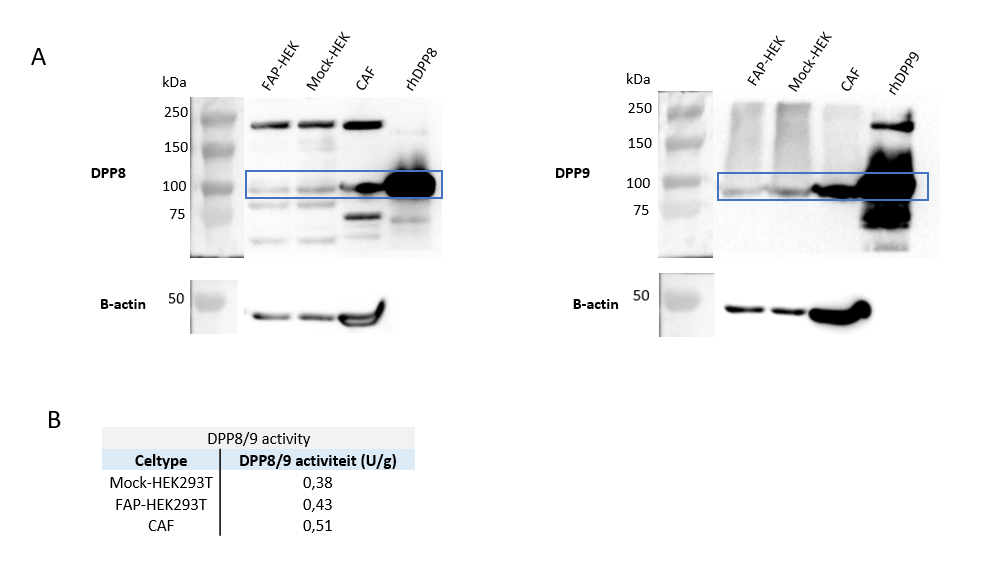
**2.2 DPP8/9 expression and activity pattern in FAP- and Mock-transfected HEK293T cells and CAFs**

Supplementary figure 3S: DPP8/9 expression and activity pattern in FAP- and Mock-transfected HEK293T and CAFs.

**(A)**DPP8 and DPP9 is expressed in all three cell lines. rhDPP8 and rhDPP9 were overloaded. The anti-DPP8 antibody is not fully selective **(B)** DPP8/9 activity is present in all three cell lines.

**2.3 Whole slide images of the urothelial cancer sections stained with the FAP-selective Cy3 and Cy5 ABP**


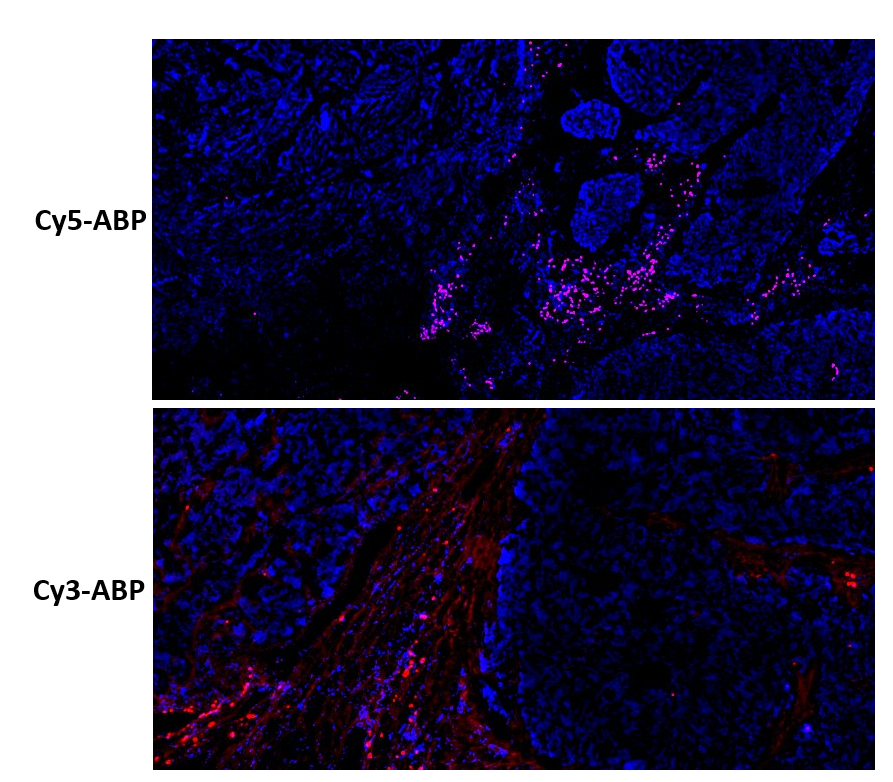


**Supplementary figure 4S: Whole slide image (WSI) of urothelial cancer section visualized with the Cy3 and Cy5 ABP**

The staining with both the Cy3 and the Cy5 probe is restricted to the tumor stroma of the urothelial cancer sections, whereas almost no staining is visible in the tumor cells themselves. Since FAP is only expressed in the stroma of urothelial cancer, it can be concluded that selective imaging of active FAP was achieved. Images are representative for *n* =3.

**3. References**

[1] Toms, J., Kogler, J., Maschauer, S., Daniel, C., Schmidkonz, C., Kuwert, T., and Prante, O. (2020) Targeting Fibroblast Activation Protein: Radiosynthesis and Preclinical Evaluation of an (18)F-labeled FAP Inhibitor, *Journal of nuclear medicine : official publication, Society of Nuclear Medicine*. doi:10.2967/jnumed.120.242958.

[2] Jansen, K., Heirbaut, L., Verkerk, R., Cheng, J. D., Joossens, J., Cos, P., Maes, L., Lambeir, A. M., De Meester, I., Augustyns, K., and Van der Veken, P. (2014) Extended structure-activity relationship and pharmacokinetic investigation of (4-quinolinoyl)glycyl-2-cyanopyrrolidine inhibitors of fibroblast activation protein (FAP), *Journal of medicinal chemistry* *57*, 3053-3074. doi:10.1021/jm500031w.

[3] Matheeussen, V., Lambeir, A. M., Jungraithmayr, W., Gomez, N., Mc Entee, K., Van der Veken, P., Scharpe, S., and De Meester, I. (2012) Method comparison of dipeptidyl peptidase IV activity assays and their application in biological samples containing reversible inhibitors, *Clinica chimica acta; international journal of clinical chemistry* *413*, 456-462. doi:10.1016/j.cca.2011.10.031.

[4] de Vasconcelos, N. M., Vliegen, G., Goncalves, A., De Hert, E., Martin-Perez, R., Van Opdenbosch, N., Jallapally, A., Geiss-Friedlander, R., Lambeir, A. M., Augustyns, K., Van Der Veken, P., De Meester, I., and Lamkanfi, M. (2019) DPP8/DPP9 inhibition elicits canonical Nlrp1b inflammasome hallmarks in murine macrophages, *Life Sci Alliance* *2*. doi:10.26508/lsa.201900313.
